# Virtual Primary Care—Improving Access, Efficiency, and Patient Experience

**DOI:** 10.1089/tmr.2024.0078

**Published:** 2024-12-09

**Authors:** Crystal Kong-Wong, Michelle Lin, John D. Scott

**Affiliations:** ^1^ Department of Family Medicine, University of Washington School of Medicine, Seattle, Washington, USA.; ^2^ Department of Digital Health Office, UWM, Seattle, Washington, USA.; ^3^ Department of Medicine, University of Washington School of Medicine, Seattle, Washington, USA.

**Keywords:** virtual care, primary care, on-demand care

## Abstract

The United States faces a growing scarcity of primary care resources within an already overstressed and poorly accessible health care system. Many health care organizations are evaluating novel models of care and adoption of digital technologies to improve primary care access and efficiency of health care delivery. This article describes a virtual primary care (VPC) model that expands access and increases the efficiency of the traditional primary care team by utilizing on-demand and asynchronous digital tools. In the first year of operations, this service line provided >21,000 on-demand telemedicine visits and >4,000 asynchronous eVisits. The VPC service line improved access to primary care services in a financially successful and sustainable model.

## Introduction

Primary care in the United States has been at the forefront of many crises faced by the American health care system: poor access, physician burnout, financial struggle, and changing consumer demands. These stressors compound on an already strained system. In the United States, only 31% of the physician workforce comprises primary care physicians despite providing 46% of medical visits.^1^ In addition, a shortage of 21,000–55,000 primary care physicians is predicted by 2032 due to decreasing numbers of students choosing to go into primary care.^2^ In recent years, leaders and innovators have looked to digital health tools as a solution for these problems.^3^ Some models have sought to completely divorce virtual services from the physical office; vendors of on-demand telemedicine services fall into this category. As the pendulum swings back, many of these companies are collapsing. In this article, we describe a sustainable, integrated, and collaborative model for virtual primary care (VPC) that uses digital tools to enhance the patient and provider experience of primary care.

## Strategic Drivers Leading to Inception of VPC

University of Washington Medicine (UWM) and the University of Washington School of Medicine (UWSOM) have long been leaders in the provision, innovation, and education of primary care for the Washington, Wyoming, Alaska, Montana, and Idaho (WWAMI) region. UWM Primary Care and Population Health (UWMPC) oversees 17 primary care clinics that provide full-spectrum primary care (adult, pediatric, obstetrical care, and in-office procedures) for UWM. The geographical reach of UWMPC extends 130 miles across the main population center of Washington state, including a clinic on the San Juan Islands. On average, in the United States, there are 111 primary care physicians per 100,000 patients; however, in the WWAMI region, this ratio is far lower. Even Washington state, with a large technology-centered economy based in Seattle, has 77 PCP MDs/100,000 people.^1^

Like many systems, UWM pivoted swiftly to virtual care during the early stages of the COVID-19 pandemic. As patients and providers adopted telemedicine visits rapidly, UWM sought to further utilize virtual tools to enhance the patient experience and organizational efficiency. Through business planning and collaboration with multiple internal stakeholders, including ambulatory and hospital leaders, IT services, the Access and Innovation team, UW Population Health, and UWM Finance, the UWM Digital Health Office (DHO) put together a pro forma for a VPC service line and implemented this in partnership with UWMPC. VPC was designed to help UWM achieve on many strategic areas:

1. Patient access—Although accessibility in health care is often defined as availability of appointments, accessibility also entails ease of access. VPC was designed to be a single place where a patient could start their care and facilitate necessary next steps, rather than having to navigate a complex and frustrating system.
2. Financial bottom line—One large barrier to increasing access is the cost associated with expansion. The VPC model allowed UWM to expand with minimal upfront overhead costs and continuing operating cost.
3. Market share—In the tech-savvy, digitally connected region of Western Washington, it was important to UWM to meet patient demand for virtual services, stay competitive, and capitalize on its reputation in the community.
4. Workforce satisfaction—Although much of health care delivery remains an in-person job, many health care workers need or prefer a remote option. VPC was an opportunity to hire new individuals and retain excellent employees.^4^

### UW VPC market considerations

When building a new service line from scratch, there were many options and opportunities to consider. One key aspect of the creative process was assaying the market landscape and surveying colleagues at other academic medical centers (AMCs) who had set up similar service lines. We found that most AMCs developed on-demand service lines out of necessity during the COVID-19 pandemic.^5^ Many VPC service lines were built to provide virtual COVID services, keeping patients out of physical clinics. As the pandemic waned, these systems looked for other ways to utilize this virtual workforce and platform, including pivoting to primary care services such as chronic disease management, virtual urgent care (UC), and bridging new patients to their upcoming primary care appointment.

It was important to understand the demographics of patients who utilize on-demand telemedicine services lines in practice. For example, many systems reported that these service lines were predominately utilized by existing patients, rather than drawing in new patients as initially intended. Another observation was that clinician bandwidth was full almost immediately after opening as a byproduct of poor primary care access.

After we evaluated market offerings and utilization patterns, the DHO began working on a pro forma. UWMPC had a wealth of experience projecting costs, revenue, and growth for opening new primary care clinics, and we were able to apply this to benchmarking costs for VPC. In addition, UWMPC had data on in-basket volumes, delay in appointment scheduling, nurse triage message volumes/themes, phone calls, and UC volume. This allowed us to project visit volumes, set a productivity target, determine staffing ratios, and put together a budget.

### Programmatic design and implementation

#### VPC offerings

We moved to define core services offered in VPC and constructed the operational infrastructure needed to support care delivery. The VPC workgroup decided to offer video visits (telemedicine [TM]) and eVisits. TM visits would be offered mostly on demand, with a small portion of the template saved for internal primary care use. eVisits are a standardized questionnaire that a patient fills out asynchronously through the patient portal. Based on their answers, a patient can receive care such as medication prescriptions or lab tests.

### TM visits

#### We defined two types of TM visits

On-demand video visits (ODVVs)—The patient accesses a queue through a patient portal. This is also available through the uwmedicine.org website. A staff member takes patients off the queue and places them on the provider’s VPC template. The provider sees the patient in the order they are placed on their template. The IT build is an out-of-the box module available in the patient portal. We were able to personalize verbiage and how the waiting queue works. Rather than allowing patients to back up in the queue, we decided to have the queue lock out additional patients when the calculated wait time got to >30 min. If the VPC queue is full, the patient gets a message asking them to check-in again in a few minutes. In our experience with brick-and-mortar UC, the online queue often has an extremely long wait time, which dissuades patients from getting care.

Scheduled TM visits—To offer some same-day/acute access to primary care clinics, we made a portion of the visits available for internal primary care clinics or triage RNs to book patients. We put careful thought into the ratio of ODVV availability versus scheduled availability and wanted to ensure that we did not use all our on-demand time for scheduled visits.

### eVisits

In planning for eVisits, first we identified the patient population who would be eligible and then worked on specific conditions.

VPC started with a COVID therapeutics eVisit that had been activated during the COVID-19 pandemic. With the availability of nirmatrelvir/ritonavir (Paxlovid), UW Medicine decided to shunt all requests for this medication through an eVisit to a team of providers responsible for pandemic-related care. With the advent of VPC, coverage of Paxlovid eVisits was transferred to the VPC team. The pandemic-era COVID-19 therapeutics eVisit was available to only established patients with MyChart accounts. We decided to continue that restriction in VPC with the addition of limiting eVisits to adults. When a provider is giving care through an eVisit, they have only patient answers and the electronic health record (EHR) to base their recommendation; thus, new patients who may have incomplete medical records pose a safety risk.

In addition, the UWMPC team reviewed EHR prebuilt content for eVisits. The primary care team decided to limit their scope to conditions that did not need specialty input. Initial eVisits included pink eye, motion sickness prevention, UTI/bladder problems, vaginal discharge, tobacco cessation, and erectile dysfunction.

### Billing and compliance

The DHO worked with the UWSOM faculty practice plan and compliance team to design billing and consent workflows for eVisits. We set up a patient portal-based consent process and built out landing pages on the uwmedicine.org website where patients could learn more. We decided to put eVisits through standard insurance billing rather than offer these as cash services. Patient portal-based self-service tools and front desk staff workflows were designed to ensure that current insurance information was collected and coverage was verified.

### Staffing

The last part of design was to detail a staffing model and workflows to support VPC. We felt strongly that VPC needed to operate like a traditional ambulatory clinic with staff to support patient flow, coordination of care, and hand offs.^6^ UWMPC-designed job descriptions for medical assistants (MAs) and patient service specialists to support this service line. We designed an Advanced Practice Provider (APP)-heavy workforce with MDs as clinical leaders and support. All the staff and providers in VPC work remotely from home; they do not have other in-person duties. Everyone also has a designated in-person location to work from if they have poor connectivity, outages, or other problems with their home workstation.

Communication is always key to a successful workplace, and it was essential to figure out a way for the virtual providers and staff to communicate. UWMPC decided to utilize the electronic health record (EHR) chat function and build out groups that comprised the people working that day. Staff workflows for coordination of patient care, such as verifying benefits, “virtual rooming” of TM patients, and coordinating lab or radiology orders, are based on in-person workflows. All staff had traditional primary care experience, and the VPC manager had previously been a supervisor at a UWMPC clinic. In addition, all providers had previous primary care experience and provided the full family medicine scope of care.

### Check-in, arrival, and rooming workflows

Common primary care patient check-in workflows were expanded to include VPC, including patient portal self-check-in and verification of benefits for TM visits and eVisits. In addition, every patient who is seen for TM gets MA virtual “rooming” to review reasons for visit, medication list, refill requests, and verification of pharmacy. The virtual MA can also help troubleshoot connectivity issues.

### Ancillary testing

Providers often order ancillary testing as part of a visit. In this case, the staff will help patients facilitate this care within the UWM system. VPC PSRs and MAs are prepared to help set up lab, vaccine, and radiology appointments. If the patient is not located with easy access to UWM facilities, the staff can also facilitate faxing orders to outside lab agencies or radiology providers.

### Scheduling and referrals

All VPC staff are trained to schedule appointments. They can set up follow-up appointments with continuity providers. In addition, the VPC staff can communicate with the primary care clinics. Complex scheduling can be a warm hand off to the patient’s home primary care clinic.

Referral scheduling utilizes the same referral processing and appointing pools that primary care uses. When a VPC provider orders a specialty referral, it automatically routes to the UWM referral team for processing and scheduling.

### In-between visit care/in-basket

The VPC services generate in-basket work such as refill requests, patient emails, and telephone calls. Staff share in the management of the in-basket and follow standard primary care procedures for routing messages, pending orders, and facilitating patient care. There is centralized support for prior authorizations, refill requests, and referral management. VPC providers rotate through the in-basket and address messages. All chart notes and patients’ messages are also copied to the primary care provider to facilitate continuity. Messages that would be better handled by the primary care team can be routed to that in-basket pool.

### Communication and visibility within UWMPC

A robust marketing, communication, and visibility campaign was provided for the primary care workforce. This included meeting with clinical and operations leaders and partnering with UWMPC leaders to ensure that awareness of this new service line was broad and deep. Emphasis was placed on communicating that this service line was fully integrated into primary care, VPC staff were available, and documentation was clearly outlined in the EHR. In addition, the VPC clinical and operations leaders were included in all typical primary care leadership meetings, creating comradery, partnership, and collaboration between VPC and the primary care clinics. The VPC providers are integrated into the provider organization chart within UWMPCPH and included in all provider meetings and events.

### Integration within AMC

It was important to make sure VPC was integrated within the greater AMC and UW Medicine. Operationally, as summarized above, all VPC workflows were congruent with UWMPC workflows, which follow standards set by UW Medicine in areas of compliance, scheduling, billing, and accessing specialty and ancillary services. In addition, a strong communication push was accomplished through communication strategies at multiple levels. During strategic business planning, design, and then rollout, UWM executive leadership, both at the enterprise and entity (hospitals and ambulatory entities) levels, were kept in the loop on strategic fit, progress, and financial results.

As we approached going live, the DHO worked on a conversation roadshow. We targeted all major operational leadership meetings, the board of trustees, and patient advisory panels. In addition, we worked with marketing on websites and internal communications to disseminate.

## Results

### Demographics

The demographic breakdown of patients using the VPC services is summarized in Table 1. Patients identifying as female were almost twice as likely to utilize VPC than patients identifying as male, with 4% of patients identifying as transgender, nonbinary, or other gender than male/female. The most common age range is 27–54-years-old, and the vast majority of patients speak English.

**Table 1.** Demographics of Virtual Primary Care Patients

July 1, 2023–June 30, 2024	ODVV		eVisit		Total
Volumes	21,618		4,228		25,826
Gender Identity					
Female	9,990	46.2%	2,226	52.9%	12,216
Male	5,386	24.9%	988	23.5%	6,374
Transgender	126	0.6%	36	0.9%	162
Nonbinary	234	1.1%	30	0.7%	264
Gender queer	141	0.7%	35	0.8%	176
Agender	17	0.1%	3	0.1%	20
Gender fluid	40	0.2%	8	0.2%	48
Other	42	0.2%	5	0.1%	47
Chose not to disclose/no data available	5,642	26.1%	897	21.3%	6,539
Race					
White	15,934	73.7%	3,201	76.1%	19,135
Asian	3,236	15.0%	622	14.8%	3,858
Black or African American	1,435	6.6%	219	5.2%	1,654
American Indian or Native Alaskan	298	1.4%	62	1.5%	360
Native Hawaiian or Pacific Islander	188	0.9%	27	0.6%	215
Another Race	262	1.2%	37	0.9%	299
None of the above	7	0.0%	0	0.0%	7
Chose not to disclose/no data available	1,364	6.0%	202	4.6%	1,566
Language					
English	21,103	97.6%	4,094	97.3%	25,197
Mandarin	60	0.3%	33	0.8%	93
Spanish	64	0.3%	8	0.2%	72
Unavailable or unknown	56	0.3%	0	0.0%	56
Russian	20	0.1%	6	0.1%	26
Korean	14	0.1%	5	0.1%	19
Vietnamese	12	0.1%	3	0.1%	15
Sign language: ASL	14	0.1%	0	0.0%	14
Farsi/Persian	7	0.0%	1	0.0%	8
Somali	8	0.0%	0	0.0%	8
Amharic	5	0.0%	1	0.0%	6
Cantonese	5	0.0%	1	0.0%	6
Panjabi/Punjabi	5	0.0%	1	0.0%	6
Tigrinya	6	0.0%	0	0.0%	6
None of the above	239	1.1%	55	1.3%	294
Payer					
Commercial	14,467	66.9%	2,859	67.94%	17,326
Medicare	3,581	16.6%	917	21.8%	4,498
Medicaid	3,060	14.2%	368	8.8%	3,428
Self-pay	374	1.7%	52	1.2%	426
Tricare	114	0.5%	10	0.2%	124
Other	14	0.1%	1	0.0%	15
Worker’s Comp	7	0.0%	0	0.0%	7
Case rate	1	0.0%	1	0.0%	2

The racial breakdown of VPC patients is majority White, with the next most common racial group being Asian, followed by African American. Patient payer mix skewed toward commercial insurers, with the next most common payer group being Medicare (Table 1).

### Turnaround time

On average, patients waited 17 min for an ODVV and 167 min for an eVisit response (80 min during working hours). The most common eVisit was for COVID-19 therapeutics (Table 2).

**Table 2.**
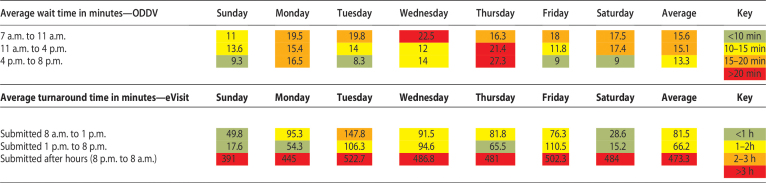
Wait Time for on-Demand Video Visits and eVisits, by Time of Day

### In-basket and between-visit work

Outside of direct patient care, VPC has generated a minimal amount of between-visit work (patient emails, phone calls, medication refills, etc). This averaged 0.42 messages per patient and 0.035 medication refills per patient seen in VPC.

### Patient feedback

There have been some interesting early trends in patient satisfaction. UWM uses the Press Ganey TM patient satisfaction survey to gauge patient feedback on TM services. All patients who have an email listed with UWM and have a TM visit get this survey via email. There are several key composites that the DHO focuses on:

1. Access
2. Ease of use of technology
3. Willingness to recommend this service

Overall, it seems like patients who utilize VPC are less satisfied with health care access at UWM, more facile with the use of technology tools, and about equivalent in general willingness to recommend the TM service.

## Discussion

VPC has been an effective and highly utilized service line at UWM. We have achieved high-volume TM with a quick turnaround time (17 min on average). eVisits continue to grow and are a convenient way for patients to request care at any hour of the day. Although most patients served by VPC are established patients, VPC has attracted new patients to the system. We can provide ODVVs in languages other than English; however, most encounters are provided in English. All payor groups have been represented, though most visits are for patients with commercial insurance.

Although a resounding success based on early results, we have identified some areas for improvement.

### Staffing

Initially, a “best guess” calculation was used to estimate staffing needs to cover the initial business hours, 7 days a week, 12 hours a day (8 a.m. to 8 p.m.). This included minimal staffing ratios for PSRs and MAs. In addition, schedules were designed to have low coverage in the early AM hours, robust staffing in the mid-morning to afternoon, and lower coverage in the evenings. This was adjusted as business picked up swiftly. In addition, UWMPC moved to add more providers and staff. By 6 months in, UWMPC had listed postings for four additional APPs, 1 MD, and an MA lead position.

### Patient experience and human-centered design

UWM went with an “out of the box” tool from the EHR that plugs into the patient portal. In user experience testing, users noted that the VPC tool looks consistent with many other items, such as “request an appointment” or “refill a medication,” getting lost in the myriad of options available in the menu. Unfortunately, there were minimal opportunities to differentiate way finding outside of standard menu selections. We have received patient comments stating that it was difficult to locate the tool in the patient portal. As a next step, the DHO is evaluating the patient experience of the patient portal and utilizing human-centered design to improve the patient experience.

Similarly, there is still work to be done integrating VPC into the typical list of options that staff and providers offer patients when they request care. Patients report that they wished someone had informed them about this service earlier or complain that their UWM clinic team did not know this service line existed.

UWM would also like to evaluate the impact that patient preference has on utilization of services. Many patients utilize VPC as an easy access alternative to waiting for an in-person visit with a continuity provider. However, if given equal access to the spectrum of services, the patient might have preferred an alternative method of receiving care. Certain virtual, on-demand services may also be more attractive to historically stigmatized groups or those reluctant to participate in health care, as these services feel more anonymous and asynchronous.^7,8^

### Next opportunities

UWM is looking at other ways to use this facile, flexible, and efficient tool. One area that VPC is delving into is the population health space. This includes chronic disease management, preventative care, and Medicare annual wellness visits. Primary care clinics have partnered with VPC to bring in patients with care gaps and difficulty getting these needs met in the traditional setting. Barriers include lack of appointment availability, inability for the patient to get time off work for an appointment, or difficulty finding childcare. VPC is a convenient way to address these issues in a way that works around the patient’s needs. UWM is also investigating ways that this service line can be marketed to value-based contracts.

The DHO is investigating how ODVVs and eVisits improve access. This includes more analysis of VPC access metrics versus primary care access but can also be applied to specialty care, for instance, eVisits for established low-complexity specialty patients. We are also considering how these tools would partner with a remote patient monitoring program or transitional care.

### Medical education

It is important to UWSOM to ensure that graduates are prepared to practice in an ever-changing health care landscape.^9^ One area where the UWSOM has expanded is into training of virtual care modalities, including TM and asynchronous care such as eConsults and eVisits. We integrated VPC into the existing virtual care delivery rotation the UWSOM offers. Students can sign up for this elective and spend some of their clinical time working with the VPC MD attendings, learning how to provide patient care through virtual means. This is also an opportunity for our virtual care providers to obtain the teaching hours necessary for promotion through the clinical professorship track in the UWSOM.

## Conclusion

As health care innovates, one clear trend is the move toward models of care that are flexible, low-cost, and allow the patient to self-serve. There are a lot of financial, access, patient satisfaction, and quality benefits to setting up these types of services. One of the keys to the success of the UWM VPC program, however, is its ties to a traditional tertiary health care system. This allows the patient to access a full spectrum of services and for the primary care team to toggle the patient on a care continuum to lower acuity or higher acuity solutions. As the cost of health care rises, patients have less tolerance for high-cost, high-effort services, and health care systems will need to set up foundational platforms, such as VPC, to be competitive and effective.

## Abbreviations Used

AMC: sacademic medical centers
EHR: electronic health record
MAs: medical assistants
UWM: University of Washington Medicine
UWSOM: University of Washington School of Medicine
VPC: virtual primary care
